# “Could you give us an idea on what we are all doing here?” the Patient Voice in Cancer Research (PVCR) starting the journey of involvement in Ireland

**DOI:** 10.1186/s40900-021-00301-1

**Published:** 2021-09-13

**Authors:** Éidín Ní Shé, Aoife Gordan, Barbara Hughes, Tom Hope, Teresa McNally, Ramon Whelan, Mary Staunton, Margaret Grayson, Liane Hazell, Iseult Wilson, Richard Stephens, Elaine Quinn, Amanda McCann

**Affiliations:** 1 School of Population Health, University of New South Wales, Kensington, Australia; 2 UCD School of Medicine, University College Dublin, UCD, Belfield, Dublin 4, Ireland; 3 UCD Conway Institute of Biomolecular and Biomedical Research, University College Dublin, UCD Belfield, Dublin 4, Ireland; 4 Expert by Experience, Prostate Cancer Patient, Dunboyne, Ireland; 5 Expert by Experience, Representing Family Carers, Dublin, Ireland; 6 Expert by Experience Testicular Cancer Patient, Dublin, Ireland; 7 Expert by Experience, University College Dublin (UCD), Dublin, Ireland; 8 Northern Ireland Cancer Research Consumer Forum, Belfast, UK; 9 National Cancer Research Institute (NCRI), UK Forum Programme Manager, London, UK; 10 School of Nursing and Midwifery, Queen’s University Belfast, Belfast, UK; 11 Consumer Forum, National Cancer Research Institute, London, UK

**Keywords:** Patient and public involvement, Cancer research, Patients, Consumer engagement

## Abstract

**Background:**

Involving patients and their carers in research has become more common, as funders demand evidence of involvement. The ‘Patient Voice in Cancer Research’ (PVCR) is an initiative led by University College Dublin (UCD) in Ireland. It encourages and enables people affected by cancer, and their families to become involved in shaping and informing the future of cancer research across the island of Ireland. Its aim is to identify the questions and needs that matter most to **(i)** people living with a cancer diagnosis, and **(ii)** those most likely to improve the relevance of cancer research. The initiative commenced in April 2016.

**Methods:**

This paper presents a reflective case study of our journey thus far. We outline three key stages of the initiative and share what we have learnt. At the core of PVCR, is a focus on building long-term relationships.

**Results:**

We have developed over time an inclusive initiative that is built on trust and respect for everyone’s contributions. This work is grounded on collegiality, mixed with a good sense of humour and friendship.

**Conclusion:**

The development of PVCR has taken time and investment. The benefits and impact of undertaking this work have been immensely rewarding and now require significant focus as we enhance cancer research across the island of Ireland.

## Background

There is a diverse and growing literature capturing the various ways of undertaking public and patient involvement (PPI) in health and social care research [1, 2, 3, 4]. Our working definition of public and patient involvement, is that it is an inclusive enabling process where research is carried out ‘with’ or by’ members of the public [2, 4]. There is evidence of changes within health charity organisations of growing and enabling public and patient involvement within their own structures [5, 6, 7]. Funding schemes have also shifted, enabling involvement in various activities such as peer reviewing applications, having members of the public as co-applicants and setting research priorities [4, 8]. In 2017, two national research funders in Ireland; the Health Research Board (HRB) and the Irish Research Council (IRC), launched a joint call entitled ‘PPI Ignite’ to support higher education institutions to embed PPI deeply into their organisational culture [4]. Recent international literature has focused on the improved integration of involvement within research settings and infrastructures [9, 10]. Although the influence of organisational contexts in supporting PPI is acknowledged as very important, it is rarely captured in the literature [11, 12]. The available evidence suggests that the key enablers to move diverse involvement from research and project specific context to embedding it within an infrastructure include **(i)** having a lead researcher who believes and champions PPI and **(ii)** a culture of PPI that is supported and celebrated as best practice [12].

This paper presents a case study of the University College Dublin (UCD) Conway Institute of Biomolecular and Biomedical Research’s commencement of their involvement journey. The institute is a multidisciplinary centre with over 400 research staff working in biomedical research and innovation. There is a strong focus on translation of this knowledge through industrial, academic and clinical partnerships [13]. “Patient voice” is a term that has become frequently used in health and social care settings. It is often used to describe a compilation of many patients’ and carers’ expressed feelings, concerns, and experiences during an illness [14, 15]. A core focus within the Institute is on cancer research. Significant work has been published on the merits and reasons for undertaking PPI in cancer research [16–20].

The motivation to involve people in Ireland is no different to international experiences. Each year in Ireland an average of 43,000 new cases of cancer are diagnosed [21]. The National Cancer Strategy 2017–2026 sets out the vision for delivery of cancer services in Ireland [22]. Central to the strategy is that services are in place to better serve the needs of cancer patients, their families and carers. The strategy sets out the need for future support services to be enhanced through a better understanding of patient needs [22].

In retrospect, the journey of the PVCR may seem quite orderly and logical, but the reality was more of an evolution through discussion, especially in the first 2 years. It is best described by a conversation between a cancer patient at the first steering committee meeting and Professor Amanda McCann. The lady asked, “*Could you give us an idea on what we are all doing here?*” to which Amanda replied, *“Quite honestly, I have no idea!”.* This paper sets out our collective experiences to date.

## Methods

This is an exploratory reflective single case study of the establishment of the Patient Voice in Cancer Research (PVCR) initiative within the UCD Conway Institute over 4 years (2016–2020). Our focus is on sharing the PVCR journey and a case study approach allowed us to share our experience within our context “without control over actual behavioural events by the investigator” [23].

### Description of the case study: the Patient Voice in Cancer research (PVCR)

**Fig. 1:**
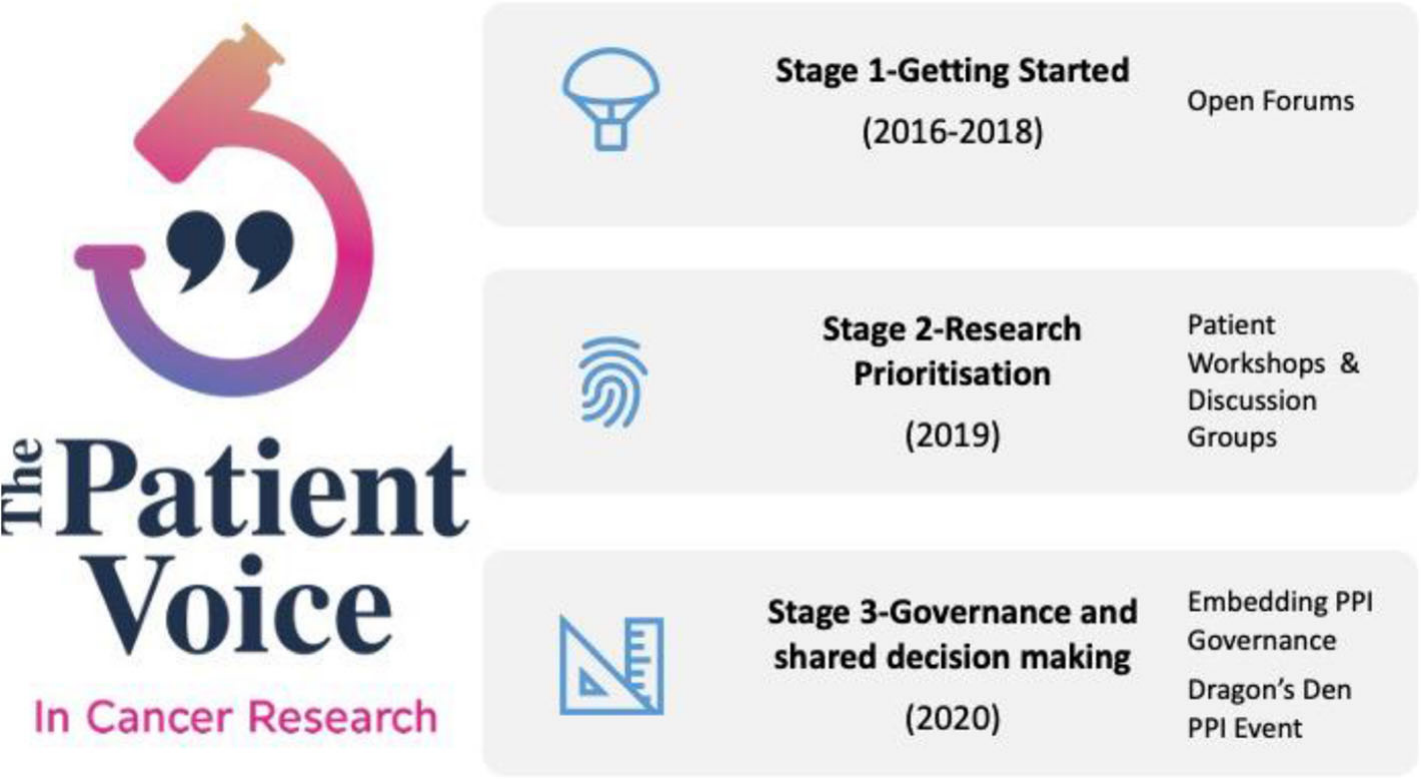
The Patient Voice in Cancer Research (PVCR) Stages, Since 2016 there have been eight PVCR cancer events **(**Table 1**)**

The PVCR commenced in 2016. The three main phases outlined in this case study cover 2016–2020 (Fig. 1).

**Table 1:** Summary of PVCR initiatives

Name	Format	Date
Placing Survivorship at the Heart of the Research Agenda	Research laboratory tours & facilitated roundtable discussion (*n* = 100)	**13–4-16**
Creating the Roadmap	Talks & designing a survey for patients to be involved in research (*n* = 65)	**19–10-16**
Is there a Clinical Trial for Me? Crowdsourcing Histopathological staining	Talks & panel discussion (*n* = 85)	**12–4-17**
Workshop 1: Fund my Research; Workshop 2; Know my DNA	Talks & interactive session on advanced diagnostics (*n* = 98)	**11–4-18**
Cancer laboratory tours - research in action	Research laboratory tours (*n* = 65)	**12–9-18**
Have your Say - National Patient Experience Survey	Facilitated workshop with the National Cancer Registry of Ireland (NCRI) (*n* = 53)	**10–4-19**
Streamlining participation in health research biobanks	Facilitated workshop with National Biobank Working Group (*n* = 49)	**9–10-19**
Dragons’ Den - Involving Patients & Carers in Research	Facilitated roundtables hosted by research groups in partnership with the National Cancer Research Institute (NCRI) UK (*n* = 93)	**25–2-20**

### Study design

The authors named in this paper contributed to sharing their reflections in a format that suited them. The write-up occurred during the Covid-19 pandemic, and we wanted to be as flexible as possible. The data used was captured in a number of ways:

1. Reflections from the authors received via email, phone conversations facilitated by two authors who were not directly involved in the PVCR (ÉNS & AG).
2. Feedback from Friendly Dragons (patient representatives and carers) *n* = 52 and researchers *n* = 10 who attended and facilitated a Dragons Den event in February 2020. Feedback was collected from both the Friendly Dragons (patient representatives) (Appendix I) and researchers immediately following the event (via paper and online forms). In addition, to preliminary feedback, researchers were also required to complete a 6-month post-event update, with the aim of capturing the impact of the PVCR/NCRI UK Dragons Den session. The outcomes from this feedback is in preparation as a subsequent publication.
3. Initial review of the materials by two authors not directly involved in PVCR (ÉNS & AG) to scope out a case study approach using Fig. 1 as the guide.
4. Initial draft of a summary shared with all authors and iteratively refined for feedback with a focus on lessons learnt.

Merging of feedback and final paper sign off by all.

## Results

### Stage 1: getting started (2016–2018)

Since the inception of the UCD Conway Institute in 2000, one of the stated goals has been to ‘build bridges to the community at large’. UCD Conway established a public engagement programme to advance our objectives under this goal and give visibility of our research programmes to the public. At the UCD Conway Institute, we believe that our research is more relevant when stakeholders are actively engaged in the process. Through engagement with patients and the public, our research can reflect their valuable perspectives and is more likely to have a greater impact [14, 17]. In 2015, researchers and public engagement professionals began working with patient advocate groups on public engagement initiatives aligned with health awareness campaigns (Movember, Breast Health Day, World Cancer Day, World Health Day, Cancer Week). This involved “pop-up” information stands and tours of the Conway Institute laboratories, to give patients and the public the ‘behind-the-scenes’ view of research in action. The research teams spoke amongst other things, about using maths to beat cancer; moving from biomarkers to post-mastectomy bras; dispelling myths around diet and supplements for cancer patients. There was a positive response to the ‘open house’ tours in particular. Participants told us that they enjoyed going ‘behind-the-scenes’ and hearing about ongoing research. Mary, a patient representative, outlines how she initially got involved:

A colleague of mine saw a PVCR event advertised and sent me an email because she thought I would be interested. At that stage, I wasn’t long back to work after my treatment for acute myeloid leukaemia, which I had been diagnosed with in 2012. I wasn’t used to being in a group with others who had similar experiences to me. I had come back to work as a “cancer survivor” and everyone was so careful of me, which was lovely because I felt very safe and looked after. But it was wonderful to be in a space with people who had been through a lot of the same experiences that I had been through. Some had come through them and some were still living through them at the time. There was a great warmth there and a great camaraderie. If you volunteer to go to something like that, you don’t go along and sit in the corner, you go because you want to be involved and be useful*.*

As an institute, we felt it was important to have greater insight into the issues that mattered most to patients and how the voice of cancer patients might be amplified within the cancer research journey to positively impact on patient outcomes. The Patient Voice in Cancer Research (PVCR) was born with an event taking place in UCD on the 13th April 2016. The organising committee comprised academic staff with active cancer research programmes led by Professor Amanda McCann with research administrative support. We adapted an equitable and collegial approach to the organisation of this event both in terms of logistics and identifying and seeking approval for small streams of funding from operational unit budgets and research grant funding. Tom, a patient representative, outlines his experience at the first event in 2016:

*I got involved with the Irish Cancer Society in 2013, when they asked me if I would be interested in going on a training course to become a Peer to Peer supporter and to share my experience with other prostate cancer patients. In 2016 the Irish Cancer Society rang me up 1 day and told me about the Patient Voice in Cancer Research (PVCR) Committee who were just starting out in UCD and asked me would I be prepared to join the committee as a patient representative. I went along to that first meeting in 2016. I was interested and got involved in it from there. When you’re retired, you’re looking for something to get involved in and be focused on. I found that first meeting to be very open and friendly. I already knew several people from other groups I had been involved in. I was among friends. I felt the objective was very open and it was a way to contribute to cancer programmes, advocacy and research.*

We retained the ‘open house’ tour aspect and followed this with a facilitated roundtable discussion forum on placing survivorship from cancer at the heart of the cancer research agenda. We define cancer survivorship’ broadly to be inclusive of the many ongoing physical, social and psychological needs of people who were living with cancer and those who survived [24]. At the time, we were unsure whether patients and their families would be willing to travel to the university and openly discuss their thoughts and concerns. We were again very pleased by the positive response. More than 100 patients and their families participated and discussed questions relating to patient information and supports as well as patient involvement in research and clinical trials. Our table facilitators included representatives from research funding agencies (Irish Cancer Society, Health Research Board); patient advocacy groups and individuals (**The Irish Platform for Patient Organisations, Science and Industry (**IPPOSI), Clinical Trials Ireland); allied health care professionals and researchers. Ramon, a patient representative, reflects on his initial feelings and how this has shifted:

*I was a bit wary in the beginning. I didn’t want us to just be token members, needed to fill the quorum. We are anything but! I even think the patients seem to be listened to that bit more. It’s good for us to be able to put a face to the researchers. It’s always said that it’s good to put a face to the patient but it’s very important for us too, we have to know who’s there fighting our corner. In the beginning, I remember sitting around a table with researchers who were talking in very technical terms – this code number and that code number. Other people around the table were nodding and I hadn’t a clue. Now my confidence has grown and I’m not afraid to interrupt them and not feel intimidated. I’ve found that by speaking out, there are always others who are on the same page as myself. Also, I find now that the researchers are much better and quicker at explaining what they are trying to say in terms I can understand. So it really has developed into a two-way dialogue.*

The report from that event (April 13th 2016) summarised the key recommendations and provided us with a basis from which to develop the PVCR. The event itself allowed us to meet patients who subsequently expressed an interest in being involved in the PVCR initiative. These individuals joined our organising committee, which at this point was evolving into a steering committee chaired by Professor Amanda McCann; sharing decision-making, defining the purpose of the grouping and organising two events per year that would try to address some of the recommendations arising from the April 2016 report.

After discussion, the steering committee devised the format of the biannual workshop events such as the examples below:

***Recommendation:*** *Protocols should be developed to seek consent from patients for the donation of tissue samples.*

**Workshop 2:** ‘Creating the Roadmap’ – initial development of survey to see if patients after a cancer diagnosis would be interested in becoming involved in research.

***Recommendation:*** *Ensure patients are aware of all relevant clinical trials ongoing.*

**Workshop 3:** ‘Is there a Clinical Trial for Me’ explained what clinical trials are, the different types of trials, how to find out about them and who do you ask.

***Recommendation:*** *Better information on cancer should be provided to the public.*

**Workshop 4:** ‘Fund my Research and Know my DNA’ - demystifying how research is funded, explaining how new technologies inform clinical decision making.

**Workshop 5:** Cancer laboratory tours coinciding with the launch of a new UCD Centre in Translational Oncology. This event gave participants an insight to research in action – an opportunity to hear about ongoing research directly from cancer researchers at their laboratory benches.

### Stage 2: establishing governance structures and funding to enable research prioritisation

By 2019, there was growing interest in the PVCR initiative with participants returning to events and promoting workshop events through their respective networks. There was also growing awareness of public and patient involvement (PPI) among the research community, primarily sparked by the efforts of the Health Research Board (Ireland) in particular [4, 6, 7, 8]. The steering committee of patients (*n* = 11), patient advocates (*n* = 2), researchers (*n* = 20), allied healthcare (*n* = 4) and professional staff (*n* = 5) now stood at 42 people, which was becoming unwieldy in relation to decision-making. From a UCD Conway Institute perspective, supporting the PVCR without a ring-fenced, dedicated budget was becoming increasingly more difficult. Initially we received seed funding from the Mater Misericordiae University Hospital (MMUH) and were approached by the Irish Cancer Society (ICS) to discuss the possibility of submitting a funding proposal. This would potentially place the PVCR initiative on a more formal footing in relation to governance and advance the establishment of a national network of patients and researchers involved in the initiative for the first time across the island of Ireland.

In April 2019, the ICS announced the success of our funding application, which enabled us to recruit a dedicated part-time resource for PVCR (Dr Barbara Hughes), and begin planning for three events to establish a national network. We established policies and procedures around governance during this 12-month period. At the heart of PVCR governance is a commitment to a shared journey by patients and researchers with open discussion and joint decision-making. This articulated purpose helped us define the terms of reference for a new PVCR steering group (Appendix II). We rationalised membership to a smaller, more agile group of 18 representatives; each invited for the purpose of bringing diverse voices to the table. This grouping comprised patients (*n* = 7), patient advocates (*n* = 2), researchers (*n* = 6), allied healthcare (*n* = 1) and professional staff (*n* = 2). A small number of specified-purpose three-person subcommittees were established to address areas of communication, information governance and network development. Each sub-committee has at least one patient involved as well as a member of the management committee to provide administrative support. The subcommittee lead provides progress updates to the steering committee at their quarterly meetings. Tom notes his experience of contributing as a patient representative:

*I get great satisfaction from contributing to something that I feel is adding to improvement in either the public perception of cancer treatment or science. In the Patient Voice meetings, you get that personal satisfaction. It’s nice going back and seeing the same people and hearing how they are getting on in UCD or at home. It’s a two-way dialogue. I think you have to enjoy it. You have to feel that your opinions are helping. I wouldn’t say that I know everything, but when I have an opinion, it goes into a pot. From that pot, policies appear and you feel you have contributed. It works very well.*

With PPI becoming an emerging area in Ireland, there was not an established framework within which academic institutions could easily involve patients in research [6, 8]. This led to us encountering a variety of stumbling blocks from on-campus parking restrictions and regulations (costs, wayfinding, difficulty parking in proximity to event location); determining and processing expenses incurred by patients for travel and caring responsibilities; and ethical and data sharing requirements. A number of third-level institutions in Ireland received funding from the Health Research Board (PPI Ignite) to establish frameworks that would enable PPI. We were fortunate to be able to work with UCD colleagues funded through HRB PPI Ignite to address these stumbling blocks and create policies and procedures with University College Dublin (UCD) to deal with them in future [6].

The UCD Conway Institute received funding from the Wellcome Trust to support capacity and development of public and patient involvement. We requested and were granted some of this funding to help address our own skills and knowledge gap in the area of PPI by working with Derek Stewart and Bec Hanley (UK PPI experts). They designed and delivered a 2-day ‘train the trainer’ workshop in August 2018 on introducing the concepts of PPI to researchers. We subsequently developed and delivered half-day introductory PPI training for researchers at different career stages (PhD, postdoctoral); the latter in conjunction with the UCD PPI Ignite coordinator. In addition to delivery for UCD-based researchers, training was delivered to Irish Cancer Society funded PhD students and postdoctoral fellows on an international training and career development fellowship programme funded by Marie Sklodowska-Curie COFUND Action (UCD TOPMed10). An important aspect of these training workshops were contributions by patients of their experience of involvement in the PVCR. We delivered communication workshops where patients and researchers would team up or ‘buddy up’ to develop plain English abstracts and engaging presentations describing research projects for patient and public audiences. This format was used for PhD students preparing for the 2019 UCD Conway Festival of Research and Innovation as well as the Professor Patrick G. Johnston Irish Association for Cancer Research (IACR) Award for Excellence in Cancer Research Outreach (2019, 2020, 2021).

The development of a brand and marketing collateral for the PVCR added to the sense of belonging and pride that was inherent to the group’s success. Mary, a patient representative who deals with graphic design companies in her professional life, secured services from a company on a pro-bono basis.

*We eventually came up with a really vibrant identity. We looked at hundreds of words that epitomised the group and whittled these down to about eight, which form part of what the Patient Voice in Cancer Research is all about. They are the ones that are epitomised in the identity. We are really happy with it now; it looks bright and inviting.*

In this stage of the initiative, there was an increased focus on collaborative approaches to setting and advancing research objectives. This led us to partner with organisations/networks such as the National Cancer Registry of Ireland (NCRI) and the National Biobanking Working Group (NBWG) to bring the voices of patients to discussions and decision-making processes relevant to cancer research. The PVCR steering committee was keen to take full advantage of such opportunities as they arose. The facilitated workshop with the National Cancer Registry of Ireland allowed patients to input to the design of a proposed national patient experience survey for 9000 cancer patients with a resulting report and National Cancer Registry of Ireland policy response paper [25, 26]. The facilitated workshop with the NBWG provided patient feedback on the design of a standardised patient information leaflet and separate consent form to streamline participation in health research biobanks. These documents must be clear and easy while also compliant with data protection regulations. They are currently being reviewed by the Irish Data Protection Commissioner.

### Stage 3: governance and shared decision-making

Toward the end of 2019, we began a discussion with the National Cancer Research Institute (UK) to co-host an event based on the successful *Dragons’ Den* concept that NCRI (UK) initiated and frequently use through their NCRI Consumer Forum [27]. At this point, there was a formal process in place for researchers or other organisations to submit a proposal outlining their idea for a patient event and how this might benefit attending patients, researchers and the wider patient network. The PVCR steering committee were very strongly in favour of the *Dragons’ Den* concept as it would bring the emphasis of the workshop event back to the core premise of facilitating discussion between patients and researchers about cancer research projects.

The event date (25th February 2020) with a location in Galway, Ireland was chosen to immediately precede the 56th Annual Conference of the Irish Association for Cancer Research (IACR), an All-Ireland, non-profit organisation for cancer researchers across all disciplines that aims to advance cancer research across the island. Fifteen research groups from around Ireland submitted proposals to participate in the event. These researchers were keen to get input from those with a lived experience of cancer (our friendly *Dragons)* on specific questions or challenges that they are faced with in cancer research projects. A sub-group of the PVCR steering committee shortlisted **ten** projects for the event with an agreement to proceed with the other five projects at a later point. Feedback was collected from both the Friendly Dragons (patient representatives) and researchers immediately following the event (via paper and online forms). In addition, to preliminary feedback, researchers were also required to complete a 6-month post-event update, with the aim of capturing the impact of the PVCR/NCRI (UK) Dragons Den session.

Overall, the experience of both the Friendly Dragons and researchers was extremely positive and demonstrated that facilitating Public and Patient Involvement (PPI) in this format, led to productive and meaningful discussion. Indeed, 96% of Friendly Dragons ‘agreed’ or ‘strongly agreed’ that their comments were listened to, and taken on board by the researchers; and felt that the feedback provided would make a positive difference to the project.

*“A great platform for patients to feel their voice matters. These inputs and experiences will hugely benefit other patients”.*

*“It felt very empowering to be part of the discussion and have a voice”.*

Similarly, the research teams that took part in the session regarded the Friendly Dragons feedback as ‘very’ or ‘extremely’ useful. This was further supported by the range of associated benefits and impacts reported by the researchers 6-months post-event.

For the majority, the feedback directly informed specific changes to their research project.

*“We have modified our patient involvement plan as a result of feedback from the Dragons…I have found the discussion hugely beneficial to our research”.*

*“The Dragons Den was highly influential in my work…the event led to an overhaul of my Interview Topic guide”.*

Even when changes were not required, researchers still reported benefitting from validation of their assumptions or approach, which led to increased confidence and motivation for the project.

*“After talking to the [Friendly Dragons] I realised the true impact that this device can have. It was very helpful for the motivation of our research team”.*

For others, the feedback informed a redirection in thinking, and an important reminder that what is deemed a priority by a researcher or clinician, may not always be a priority for the patient. Consequently, this stresses the importance of involving patient representatives at all stages of the research process to ensure the outputs are both meaningful and relevant.

*“Previously we had spoken to surgeons and oncologists, but the patients’ perspectives were very different”.*

*“I always thought I was thinking about the patient perspective, but doing the Dragons Den I found out what the patient perspective actually was”.*

The PVCR/NCRI Dragons Den event empowered both patient representatives and researchers and demonstrated that anyone with a lived experience of cancer can contribute to research when given the appropriate platform and support to do so.

## Discussion

### What have we learnt?

Since the beginning of the PVCR initiative, feedback from patients has been central to our learning. Taking time as a group to reflect after each event, and capture our collective experiences is crucial. As we chronicled our journey here, a few clear lessons should be noted.

### Adapting to digital engagement

The COVID-19 pandemic has brought changes to the way the PVCR operates. PVCR steering committee meetings are now held virtually on the Zoom platform. We have found that connecting virtually has allowed people to be involved from the comfort of their homes, saving time, expense and the inconvenience of travelling long distances. We have also held four virtual *Dragons Den* events; each with a research group, patients from the steering committee and those who have been involved in our events previously and the PVCR administrative team facilitating proceedings.

### Ensure an ongoing feedback loop and make changes

Feedback to all workshop attendees is crucial. It closes the circle and is a tangible acknowledgement of the unique contributions that each attendee has made. Feedback enables all contributors to know how much their unique input was valued, listened to and taken on board.

Participants were always very appreciative and enjoyed the informative and social aspects of the workshops we facilitated. However, quite rightly, it was noted by some that while there was a great buzz around the workshops, people left them feeling; *“yes, that was interesting”,* or *“that was well worth giving my time up for”* but there was no immediate action taken or follow through. We refined this and provided summary feedback for sessions.

Feedback was also instrumental in guiding us on the timing of our events. Initially, our workshops would have started at 9 am. This made it difficult for attendees to reach us without an overnight stay. Moreover, depending on a person’s current health and medication regime, starting later in the day is more feasible. We changed the start times of events to commence after 10 am and finish in the early afternoon to enable more patient representatives to attend. Technological responses to deal with COVID-19 pandemic restrictions has advanced the option of digital engagement. This is something we will continue with in the future and undertake more hybrid/blended events.

### It takes time to build relationships and trust

We knew that there was a genuine need and enthusiasm from PPI partners and researchers to continue the dialogue that had commenced in our tours of the cancer research laboratories in 2016 at UCD. We wanted to continue that dialogue. This was a new and uncharted environment for all of us, with many of us meeting for the very first time and therefore by default at the start of building up a relationship. Like all relationships, it could only continue with trust and respect, on an equal footing for all; starting with getting to know each other and having the sense that this was for the long haul and not just a one-off effort. Honesty was crucial. There was some naivety at the start of this initiative and that was openly acknowledged. It made us want to support each other and move the project forward and help in its development and formation.

It should be noted that it took PVCR a considerable amount of time to work out what we were going to be able to accomplish. It is testament to our PPI partners that their loyalty and commitment to PVCR gave so much support here. Experiences with cancer are so broad and unique. We began with an open call for interested parties to join PVCR.

### Support education and develop a shared language for understanding

Listening is critical to facilitate shared language and understanding. At the beginning, there was time allotted for listening, sharing individual stories and building a trustworthy relationship. When the PVCR committee first met, the cancer researchers at the table gave their name, said who they were, and shared their cancer research focus with the group. For our patients, they gave their name and the type of cancer they had. For a long time, we did not know the occupations of our patients and did not ask. On reflection, this was good as cancer may have determined that they might not be able to work in their respective occupational fields again. They were sharing with us the information that they would like us to know and in the early stages this was with people that they had never met before, whether it be researchers or other patients. The shared language therefore was a shared understanding that there was a mutual interest, a sense of a new journey and a sense of really facilitating communication but listening carefully throughout.

### Getting the governance right

PVCR started with no structure or governance to speak of. This we found in hindsight was fortuitous as it enabled our PPI partners could shape it from the start. We had no idea what we were doing at the beginning with the crucial exception that we knew the absolute importance and immense impact of bringing researchers and patients together to discuss cancer research and to uniquely identify how this research could be tailored to improve outcomes. As time went on and due to the non-prescriptive nature of the agenda, we, as a group, developed a relationship with the concept of PVCR and worked hard on our identity and structure; describing what defined us and how we would describe ourselves to others who were interested in our intent. This could never have happened if at the very outset we already had a governance structure laid down. Our governance and mission statement came together in 2019, 3 years later when clarity and purpose had been chiselled and honed into a vision equally shared by all in PVCR. A decision had to be made to tailor our committee to be, as far as possible, a truly composite committee that could reflect the voices of patients as broadly as possible. One could say it was a *co-designed* governance which needed time to get right.

### Parking, accessibility and logistics

University College Dublin (UCD), is a vast campus, and parking and accessibility are a huge challenge, especially when hosting large patient events. Due to parking limitations, attendees who had in some cases taken annual leave or planned a half day, gave up trying to find parking and left campus in frustration. We worked with Estate Services in UCD, to allocate some carpark spaces for our external attendees. We ensured that these spaces were very close to our events to ensure accessibility. For our larger events, PVCR ambassadors/volunteers would line the path to where the event was taking place and sometimes strategically positioned signage and/or balloons, were used to assist in this.

Accessibility access was always detailed clearly ahead of our events. Initially, we had not appreciated how important having a quiet room was. An attendee at an early event became unwell and needed a space to breathe, compose themselves and talk with someone as needed. There are emotions and circumstances that are unique to everyone that may be inadvertently triggered, that one can never prepare for in advance. A quiet room, a space to move away and a person to talk with were all made available at subsequent events.

### Celebrating success

Feedback to our workshop attendees, with details on how the workshop has contributed to a questionnaire, to a policy document that will impact on cancer care, or to a cancer researcher’s grant application/public and patient involvement section, is a tangible way of celebrating success. Seeing patients who were with us from the very start of the PVCR journey become facilitators, scribers, and co-applicants on grant applications is wonderful and something to acknowledge and celebrate. During COVID-19, our zoom meetings deal with the business in hand and then we always share a virtual tea, coffee and social chat for up to an hour after the committee meeting; our token “Green Room”. In relation to payment for our partners, at a minimum, all out of pocket expenses should be covered for members of the public or patients to facilitate their involvement in research. There is no recommended set payment rate but depends on the project, funding available and the individual circumstances of the involvement. We use a guidance document on public and patient involvement / public engagement costings developed by the PPI Ignite UCD team. The costings are benchmarked against those used by the National Women’s Council of Ireland and the Disability Federation of Ireland.

### Developing links across Conway, UCD and externally

There has been incredible support from the University, with the caveat that everyone is working voluntarily for PVCR. External funding from the Mater Foundation in the Mater Misericordiae University Hospital (MMUH) (2018–2019) and the Irish Cancer Society (ICS) (2019–2020) allowed a significant possibility to bring PVCR out of Dublin and into the rest of the island of Ireland. This funding was instrumental. We developed links with other areas of research in the UCD Conway Institute, to share our learning, and established a patient voice learning network within the UCD Public and Patient Involvement Ignite programme.

### The ‘patient voice’ is important in the research process

The PVCR initiative grew from an instinctive belief by researchers and research support professionals that facilitating dialogue with patients could positively impact on the research process. Now, 5 years on, patients themselves are fully affirming the importance of having their voice heard as part of the research process. Patient representatives, Mary and Tom give their perspective:

*“For a long time during my illness, I didn’t even feel that I had a voice because I was very ill and weak. I have heard researchers come along and talk about what they are trying to achieve and you know, they really need patients to talk to them and share their experiences and that has been a real eye opener.* “*(Mary).*

*“At 72, I know a lot more about life and diseases and how different events impact on your life than I did at 20. A lot of the researchers I have met are very intelligent and very young. They might not have the perspective or experience that a patient or survivor does.” (Tom).*

### Moving the dial to ‘engaged research’

The single biggest barrier or fear expressed by both patients and researchers in this PVCR journey is that the process of involving patients in research would be viewed as tokenistic. As patients and researchers work together in partnership during the research lifecycle, it is clear that involving those who stand to gain most from research outcomes is hugely beneficial. Patient representatives describe the potential impact of this type of engaged research:

*“I don’t think the patient should take over. It is important to be involved in steering a project though. The researchers need to hear the patient experience to know which direction to go in. With the chemotherapy I had, all my hair fell out which wasn’t a big deal for me or for most guys I’ve talked to. But hair loss is a huge deal for most women. So, if a researcher is dealing with testicular cancer, focusing on a side effect other than hair loss would be more beneficial. I think that’s where the patient voice comes in. Even if you’re only getting a fraction of the population’s experience, it’s still very helpful.” (Ramon).*

*“Prior to my involvement, I thought researchers were in a glass bubble, that they were very special and that they did things that nobody questioned. Now, I have learned that they are out there, craving information from patients. They want to know “What effect does this have?”, “How does this affect you?”, “What do you feel and think about this?” They need all of that to feed back into their work so that there is that subjective slant to their research. I never knew that was there.” (Mary).*

*“I have been involved in one or two projects with researchers where we help them translate their grant application into layman terms. That was the thing I felt I could help most with. I can’t help with the science of it, but I can help them restate what their objective is in layman’s terms. I am an accountant by trade. I know that accountants talk in accounting terms. The same is true in science and medicine. In a lot of cases, I felt I was able to help the researchers to get the right terminology. Sometimes you can throw too much information at somebody and it loses its relevance.” (Tom).*

## Conclusion

At the core of PVCR is a focus on building long-term reciprocal relationships between patients and researchers. In this endeavour, it is crucial to improve the reporting of public and patient involvement in research [28] (Table 2 in Appendix III). We have developed over time an inclusive initiative that is built on trust and respect for everyone’s contributions whether they be positive or negative. This work is grounded in collegiality, and a good sense of humour and friendship undoubtedly helps. The ongoing development of PVCR is time-consuming and does require investment and flexibility. The benefits of undertaking this work cannot be ignored as we enhance cancer research across Ireland.

## Data Availability

Yes

## Appendix I [Patient Representative Feedback]

**PVCR/NCRI (UK) Dragons’ Den Summary Report.**

**PVCR/NCRI (UK) Dragons’ Den** Tuesday, 25th February 2020 Galway Bay Hotel.

**Gender distribution of respondents.**

**Table:**

Male	Female	Not stated	Total
15	23	15	53
28.3%	43.4%	28.3%	100%

**Question 1. How did you hear about the DD?**

**Table:**

How	How many times?
Word of mouth	10
No response	8
Online/social media	7
Advocate/support group	6
PVCR	5
TV/Nationwide	4
IACR	3
NUIG	3
Email	3
Healthcare professionals	2
HRB	2
‘Science on Screen’	1

***Note:*** *In some cases, participants reported more than one method of hearing about the event and all have been recorded separately*

**Question 2. Indicate your Dragons Den table.**

**Table:**

Table	No. feedback forms received
1	7
2	5
3	6
4	4
5	3
6	6
7	8
8	6
9	4
10	4

**Question 3. 1. I was clear about what the DD session was before the event.**

**Table:**

Preference	No.	%
Strongly agree	18	34.1
Agree	24	45.3
Neither agree/disagree	5	9.4
Disagree	4	7.5
Strongly disagree	2	3.7

***Note:*** *In one case, the participant responded to all questions with ‘strongly disagree’ but all associated feedback was very positive. It has been recorded as indicated by the participant in Q3.2, 3.3, 3.4 and Q5.1, 5.2*

**Question 3. 2. The venue for the DD was suitable.**

**Table:**

Preference	No.	%
Strongly agree	35	67.3
Agree	14	27
Neither agree/disagree	1	1.9
Disagree	1	1.9
Strongly disagree	1	1.9
No answer	1	0

**Question 3. 3. The number of friendly dragons at my table was suitable.**

**Table:**

Preference	No.	%
Strongly agree	32	61.5
Agree	17	32.7
Neither agree/disagree	0	0
Disagree	1	1.9
Strongly disagree	2	3.8
No answer	1	0

**Question 3. 4. The session length was suitable.**

**Table:**

Preference	No.	%
Strongly agree	19	36.5
Agree	22	42.3
Neither agree/disagree	3	5.7
Disagree	7	13.5
Strongly disagree	1	1.9
No answer	1	0

**Question 3. 5. I knew who to contact if I had questions about the DD.**

**Table:**

Preference	No.	%
Strongly agree	30	56.7
Agree	20	37.7
Neither agree/disagree	2	3.7
Disagree	1	1.8
Strongly disagree	0	0
No answer	0	0

**Q4. Additional comments/suggestions- synopsis of recurring themes.**

**Table:**

Perhaps **too many at table**	
Communication pre-event was great. A very wide range of voices. Real commitment to patient voice	
**More advertising** where you host meetings coming up to event eg. local papers, hospitals, cafes	
Would like to get **more information on the other tables**	
May be an idea not to have tables but chairs around flip chart as **difficult to hear** with number of groups in room. Without the table, we would be nearer to the researcher	
**More time** would have been useful. It takes some time to get into the subject and warm up	
Would love **follow up from research team**	
Its good to meet other people living with cancer. Set up and venue excellent	
I love Amanda’s little bit of wit	
Thank you for organising this wonderful event. Amazingly beneficial to all of us out here!	
Exceptional organisation and facilitation. There was a great tone set for the whole night	
Keep up the good work. During my journey I said ‘I don’t want sympathy, I want positivity’	
Such a lovely atmosphere in the room and the whole set up was excellent - makes it easy and comfortable to talk about cancer	

**Question 5. 1. Your comments were listened to and taken on board.**

**Table:**

Preference	No.	%
Strongly agree	31	65
Agree	15	31
Neither agree/disagree	1	2
Disagree	0	0
Strongly disagree	1	2
No answer	5	0

**Question 5. 2. The feedback your group provided will make a positive difference to the project.**

**Table:**

Preference	No.	%
Strongly agree	32	63
Agree	17	33
Neither agree/disagree	1	2
Disagree	0	0
Strongly disagree	1	2
No answer	2	0

**Question 6. Did DD meet your expectations?**

**Table:**

Yes	No	No answer	%
17	0	6	100

***Note:*** *Only 17 participants answered this question directly*

**Question 7. Did you feel there was enough opportunity for questions and discussions?**

**Table:**

Yes	No	No answer	% yes	% No
27	21	5	56	44

**Question 8. Suggestions to improve DD – synopsis of key suggestions.**

**Table:**

Have a really **simple overview of the project questions** as not everyone pre reads info	
**More information** in advance	
**More time**	
Specific protected **time at start of session** to give an overview of research to dragons	
**Give more time for discussion**	
More time and no tables, just chairs around a flipchart to **hear better**	
Perhaps a **meet and greet with the researcher** where they can present their study/research in a more formal way before the discussion (separation of the two parts slightly)	
To be able to **give input to the other topics** - maybe a questionnaire emailed. Ask which topics people would like input on and email them a questionnaire	
Not sure how this could happen due to **layout** and different tables all talking at once but if one could **hear better** what is being said during discussions	

**Question 9. Selection of best quotes summing up the experience (subjective!)**

**Table:**

Fantastic opportunity to positively influence researchers’ ideas and thinking	
Good atmosphere promising positive change	
So nice to have my voice heard	
If my contribution can help one person, I am happy.	
An insightful, eye opening and thought-provoking evening	
A great platform for patients to feel their voice matters. These inputs and experiences will hugely benefit other sufferers	
Very relaxed and informal. I enjoyed meeting other people	
Empowering and creative opportunity for patient involvement	
Excellent opportunity to interact with like-minded people	
Regardless of your association with cancer, everyone has something to offer	
Excellent day - empowering patients, clinicians and researchers	
A step forward in democratising patient care	
It was a brilliant event. It felt very empowering to be part of the discussion and have a voice	
Progressive, informative and providing patients with a chance to participate in the future of cancer research	
An invaluable discussion with open minded people from a diverse background. Excellent fun!	

## Appendix II

**Patient Voice in Cancer Research (PVCR) Steering Committee.**

**Terms of Reference.**

1. Mission

The Patient Voice in Cancer Research (PVCR) Steering Committee is responsible for an initiative to actively engage cancer patients, cancer researchers and other interested parties (patient advocates, families, carers, healthcare professionals, policy-makers and those with an interest in cancer research) in discussions and decision-making processes, which positively impact on cancer research and outcomes for patients.

2. Functions

2. The PVCR steering committee, will

- Build connections between patients and cancer researchers.
- Facilitate a *two-way dialogue* between patients and cancer researchers.
- Provide education and training for patients and cancer researchers to:
  - (i) Improve communication between both parties ensuring that cancer research is seen as a joint venture to overcome the disease and improve quality of life and overall survivorship during and post treatment.
  - (ii) To improve the understanding of cancer research in a concise and clear message to the wider community.
- Inform policy-makers and healthcare professionals of research needs that will benefit cancer patients.
- Provide greater clarity to patients on the capture, collection, management and use of data in cancer research.

2.2 The PVCR steering committee may form such and as many sub-committees as it deems necessary to perform its functions, and may delegate any of its functions to a sub-committee. This list is not exhaustive but initially includes: communications, information governance, community network and training sub-committees

3. **Composition**

**At a minimum,** the PVCR Steering Committee will include:

▪ 6 patients (balance with respect to gender, age, socio-economic background and cancer type)

▪ 1 patient advocate group representative

▪ 1 representative from each registered charity

▪ 1 social science allied health professional (nursing, clinician, psycho-oncologist)

▪ 2 research active academics

▪ 2 early career researchers (up to seven years out of their PhD, as per ERC guidelines)

4. **Membership**

4.1 The Steering Committee will consist of a minimum of 15 and a maximum of 25 core members, with a quorum of 13.

4.2 Membership will be for 2 years in order to allow adjustments as required.

4. 3 Members are free to withdraw from the Steering Committee at any time by contacting the Chair.

4. 4 The Chair will be appointed by the Steering Committee for a 3-year period, after which time a new Chair or a second term of office will be discussed.

4. 5 People with specific expertise or specialised knowledge can be invited to PVCR meetings on request through or by the Chair.

4. 6 New members can be nominated by Steering Committee members, and must complete an application form which is available by emailing patientvoicecancer@ucd.ie

5. **Meetings**

5.1 Steering Committee meetings will be held 3–4 times a year, usually in advance of PVCR events to facilitate planning.

5.2 The Steering Committee will be provided with a meeting agenda and supporting papers in good time before the meeting. To avoid unnecessary burden, the paperwork will be concise.

5.3 Detailed minutes and actions arising will be circulated to the Steering Committee following meeting.

5.4 Steering Committee members who are patients will be reimbursed for travel and reasonable expenses to attend PVCR meetings in accordance with the guidance from UCD Bursar’s Office and the PPI Ignite team. As a public sector body, expenses claimed are subject to public scrutiny by the Comptroller & Auditor General and through Freedom of Information requests. It is important that all expense claims submitted should be exemplary in terms of compliance with procedures, accurate, easy to review and clearly justifiable. The relevant forms are available by emailing patientvoicecancer@ucd.ie

5. 5It is anticipated that PVCR members representing external agencies will claim reimbursement for travel expenses from their employer.

6. **Reporting**

The Steering Committee will report to funders, as per the terms and conditions of any grant received.

**7. Partners**

7.1 University College Dublin (UCD) provides support to the PVCR in its initial stages.7.2 Fudge Creative provides support in terms of branding and identity.7.3 The Irish Cancer Society approved a grant of €30,000 for one year of operation (May 2019–May 2020), to be reviewed on completion.7.4 The Mater Foundation has provided a grant of €20,000 for PVCR events 2018–2020.

## Appendix III

GRIPP2 SHORT FORM.

Staniszewska, S., Brett, J., Simera, I. et al. GRIPP2 reporting checklists: tools to improve reporting of patient and public involvement in research. *Res Involv Engagem* **3,** 13 (2017). 10.1186/s40900-017-0062-2

1. **Aim (Report the aim of PPI in the study):-**

To encourage and enable people affected by cancer and their families to become involved in shaping and informing the future of cancer research across the island of Ireland. Its aim is to identify the questions and needs that matter most to **(i)** people living with a cancer diagnosis, and **(ii)** those most likely to improve the relevance of cancer research.

2. **Methods (Provide a clear description of the methods used for PPI in the study):-**

This is an exploratory reflective single case study of the establishment of the Patient Voice in Cancer Research (PVCR) initiative within the UCD Conway Institute over four years (2016–2020). Our focus is on sharing the PVCR journey and a case study approach allowed us to share our experience within our context “without control over actual behavioural events by the investigator”.

3. **Study Results (Outcomes:- Report the results of PPI in the study, including the positive and negative outcomes):-**

**Positive.**

- UCD Conway has established a PPI programme and given visibility of our research programmes to the public that is branded and trusted.

- Biannual workshop events (*n* = 8) themed according to patient interest have been successfully hosted pre-Covid-19. During the COVID-19 pandemic, the DD events have successfully moved to the virtual environment.

-Specifically in relation to the PVCR Dragons Den (DD) events, these have involved circa 150 “experts” and to date 15 cancer researchers, in focused discussions on what researchers need expert advice on and insight on. Feedback (3 month and 6 month) from researchers who have requested and hosted these DD events, have been most positive of the impact these unique discussions have had, on the way they have re-thought certain aspects of their original research plan, and howthey have incorporated new ideas from the experts. Importantly, in some cases, these unique contacts have become team members in co-designing cancer research projects moving forward. The specific outcomes of the feedback collected is currently in preparation for a subsequent publication.

-Our Governance and Terms of Reference were ***co-designed*** with our “Experts by Experience”.

-Through PVCR we have established training for investigators in PPI, and routinely deliver communication workshops where patients and researchers team up to deliver plain English Abstracts.

-PVCR has worked with organisations for example the National Cancer Registry of Ireland (NCRI), the National Biobanking Working Group (NBWG), and the National Cancer Research Institute (NCRI) UK, to bring the voices of patients to discussions and decision-making processes relevant to cancer research.

-Feedback to attendees for all events is crucial and indeed critical.

**Negative.**

-At one of our events, one of attendees unexpectedly, became quite distressed. Since that time our events have professional psychological support available as required. In face to face events this is the identification of a room where a private discussion can be had and if online a bespoke breakout room for a private consultation.

4. **Discussion and conclusions (Outcomes-Comment on the extent to which PPI influenced the study overall. Describe positive and negative effects.**

-There are positive impacts to holding virtual PVCR events, as it negates the issue of travel, finding the location of the event and indeed logistics of parking. Moving forward, blended /hybrid approaches will be considered in PVCR events.

-Ensure an ongoing feedback loop. Feedback enables all contributors to know how much their unique input was valued, listened to and taken on board.

5. **Reflections/critical perspective (Comment critically on the study reflecting on the things that went well and those that did not so others can learn from the experience)**

**Things that went well:-.**

The patient Voice in Cancer Research (PVCR) is a recognisable and trusted brand for patients to feel this initiative as inclusive of all voices of “*experts with experience*” in a trusted and collaborative environment. Patients who have engaged with PVCR events frequently join us for a range of other events we host and indeed many of our new members across the island of Ireland have been encouraged to sign up for PVCR circulars and newsletters and indeed events based on “*word of mouth*”.

In relation to the DD events specifically, researchers have benefitted from these DD events from the perspective of learning the critical importance of including patients in their cancer research endeavours moving forward, which by its very nature needs such a multi-disciplinary team approach for cancer research to make a continued difference for patients with this diagnosis. For some researchers, partnerships from these DD events have resulted in collaborations and joint research applications co-designed by researcher and patient.

For patients, from feedback we have been given, that sense of contributing and helping researchers with their research questions has been most empowering. The fact that researcher reports are circulated back to the DD attendees has been very well received as patients have a tangible sense of how their contribution has made a difference. There is also a key sociable aspect to these DD events which particularly during COVID-19 has been very important.

**Things that did not go so well and which have now been improved:-**

1. Our start time and end time for any of the PVCR events is now tailored to times that are easier for patients to attend, particularly if travelling long distances (Pre = COVID-19).
2. Initial events lacked structures feedback to attendees in relation to their unique contribution to the discussions in hand. Feedback is now integral and crucial to every event we host.
3. A quiet room, a space to move away and a person to talk to are now available at all of our events should an attendee become unwell or distressed.

## Abbreviations

HRB: Health research board
IACR: Irish association for cancer research
ICS: Irish cancer society
IPPOSI: Irish platform for patient organisations, science and industry
MMUH: Mater misericordiae university hospital
NBWG: National biobanking working group
NCRI: National cancer registry of Ireland
NCRI: National cancer research institute
PPI: Public and Patient Involvement
PVCR: Patient voice in cancer research
UCD: University college Dublin
